# Assessing the role of eosinophil‐mediated immune response markers in detecting hookworm infection: A case‐control study in Kintampo, Ghana

**DOI:** 10.1002/hsr2.674

**Published:** 2022-06-03

**Authors:** Samuel A. Sakyi, Benjamin Amoani, Stephen Opoku, Lawrence Dzata, Wilfred Aniagyei, Ebenezer Senu, Kwabena Dankwa, Michael D. Wilson

**Affiliations:** ^1^ Department of Molecular Medicine, School of Medicine and Dentistry Kwame Nkrumah University of Science and Technology Kumasi Ghana; ^2^ Department of Biomedical Science, School of Medicine and Dentistry University of Cape Coast Cape Coast Ghana; ^3^ Department of Microbiology and Immunology, School of Medical Sciences University of Cape Coast Cape Coast Ghana; ^4^ Parasitology Department, Noguchi Memorial Institute for Medical Research, College of Health Sciences University of Ghana Legon Ghana

**Keywords:** eosinophil cationic protein, eosinophils‐mediated immune response, eotaxin (CCL11), hookworm infection, immunoglobulin E

## Abstract

**Background and Aim:**

Human hookworm disease caused by *Ancylostoma duodenale* and *Necator americanus* is a serious public health problem. Hookworm infection activates eosinophil‐mediated tissue inflammatory responses, involving the production of the eosinophil‐specific chemokine (eotaxin), recruitment of eosinophils, secretion of the cationic protein, and production of antiparasite immunoglobulin E (IgE). We investigated eosinophil‐mediated immune response as markers (CCL11, eosinophil cationic protein [ECP], and IgE) for detecting hookworm infection.

**Methods:**

This case‐control study was carried out in hookworm endemic areas within the Kintampo North Municipality.Forty hookworm‐positive subjects and 36 apparently healthy individuals were recruited as cases and controls, respectively. Stool samples were collected for hookworm detection by the Kato–Katz technique and speciation by polymerase chain reaction. Approximately, 5 ml of intravenous blood was used to obtain plasma for the immunological assays.

**Results:**

Of eosinophil‐mediated immune response markers studied, ECP and CCL11 were significantly higher among hookworm patients compared to controls. Increasing CCL11 (*β* = −0.81, *p* = 0.015) was associated with a significant decrease hookworm intensity. However, increasing eosinophil count (*β* = 0.62, *p* = 0.027) was associated with significant increase in hookworm intensity. In receiver operator characteristics analysis, ECP could significantly detect hookworm infection with a very high area under the curve (AUC) (AUC = 0.97, *p* < 0.0001). At a cutoff of 39.05, ECP was the best eosinophil‐mediated immune response marker for detecting hookworm infection with a sensitivity of 97.2%, specificity of 87.8%, a positive predictive value of 89.7%, and a negative predictive value of 96.6%.

**Conclusion:**

ECP best predicts eosinophil‐mediated immune response for detecting hookworm infection, while CCL11 and eosinophil count better predict the intensity of hookworm. Moreover, the ECP level is a good indicator of hookworm infection and intensity and may require additional investigations to augment current hookworm diagnostic techniques.

## 1 INTRODUCTION

Hookworm disease caused by the nematode parasites *Necator americanus* and *Ancylostoma duodenale* is a common helminthic infection. Globally, hookworms infect an estimated 576–740 million people.^1^, ^2^ To reduce soil‐transmitted helminthiases, a thorough and well‐timed review of epidemiological studies using sensitive and precise diagnostic techniques is needed. Lustigman et al.^3^ suggested that implementation of interventions, monitoring, and evaluating their effectiveness will help in detecting anthelminthic resistance at the early stage.^3^, ^4^, ^5^

Hookworm infection activates eosinophil‐mediated tissue inflammatory responses and involves the production of the eosinophil‐specific chemokine (eotaxin), recruitment of eosinophils, secretion of eosinophil cationic protein (ECP), and the secretion of anti‐parasite immunoglobulin E (IgE).^6^, ^7^ The synergistic effect of these mediators protect the host against the hookworm parasites.^8^, ^9^

Eosinophils are granulocytic leukocytes, which duty is in host defense and tissue pathogenesis triggered by helminth infection.^10^ In parasitic infections, eosinophils are increased and recruited into inflamed tissues under the guidance of Th2 cell‐derived cytokines and chemokines.^11^ These Th2 immune responses, culminate in IgE production and eosinophilia. IgE has been linked with protection against an extensive range of helminth infections and believed that IgE and its receptors support counter metazoan parasites.^8^

Eosinophils possess many cell surface receptors for cell signaling linked with a respiratory burst, chemotaxis, adhesion, degranulation, or apoptosis,^12^ these may be linked with eosinophil‐mediated tissue inflammatory reactions in parasitic infection. Even though many infections are linked with eosinophilia, the existence of eosinophilia in an individual with tropical exposures indicates the likelihood of particular parasitic infections. Thus, eosinophilia as a helminth infection marker requires attention and could serve as an indication to aid in diagnosis.

Eotaxin (CCL11) is a vital specific eosinophil chemokine linked with the accumulation of eosinophils at sites of infection. It is also produced in the lungs of asthmatics and functions in directing eosinophils at inflammatory sites.^13^ In addition, eotaxin is involved in the discriminatory recruitment of eosinophils into inflammatory areas in the course of parasitic and allergic reactions. Similarly, most soil‐transmitted helminthiases are driven by type‐2 (Th2). Ivanovska et al.^14^ observed high levels of eotaxin in neurodegenerative and psychiatric conditions^15^; however, no study has assessed whether eotaxin can be considered a marker for hookworm infection.

Eosinophil granulocytes produce an effective cytotoxic protein called the ECP which functions in host defense against helminth infections.^16^ The presence of helminth or an allergen can lead to the release of ECP.^17^ Subsequently, a high worm load can result in eosinophilia and hence a surge in circulating ECP levels. It is, thus, imperative to evaluate the diagnostic accuracy of ECP levels and its relation to egg counts in the milieu of the eosinophil‐mediated immune response.^18^ While the exploration for a cost‐effective, sensitive, highly‐specific, noninvasive diagnostic test for hookworm is imperative, it is important to study all products of eosinophil‐mediated immune response markers (CCL11, ECP, and IgE).

Studies directed at eosinophils have gained importance in recent times. However, no study has assessed products of eosinophil‐linked tissue inflammatory reactions in hookworm infection. The current Kato–Katz method is saddled with low sensitivity, especially for the identification of low‐intensity parasitic infections.^17^, ^19^ Against this background, the current study assessed products of eosinophil‐mediated immune responses (CCL11, ECP, and IgE) as potential diagnostic markers for hookworm infection. Identification of a sensitive correlation between these potential biomarkers and hookworm intensity will augment current diagnostic methods.

## 2 MATERIALS AND METHODS

### 2.1 Study site

The study was conducted in widespread communities within the Kintampo North Municipality (KNM) in the middle belt of Ghana. The KNM has a population of about 140,000 and 32,329 households with a total area of 7162 km^2^. The residents are mostly subsistent agriculturalists of livestock and crop.

### 2.2 Study design and sample processing

This case‐control study was conducted in the KNM. Community engagement was done through a durbar to explain the purpose and the nature of the study. Consenting community members were all screened for parasitic infection, chronic, infectious, and allergic infections. Forty hookworm‐positive subjects who fulfilled inclusion criteria were recruited as cases, while 36 apparently healthy individuals without any parasitic infection were recruited as controls. Skilled field assistants administered structured health questionnaires, and shared labeled stool containers with the participants. Stool samples were obtained and processed for hookworm detection by the Kato–Katz technique and polymerase chain reaction(PCR).^20^ Approximately 5 ml of blood was obtained by venepuncture into vacutainers containing ethylene diamine tetraacetic acid containers. The blood sample was centrifuged, and the plasma was stored at −80°C until ready to be used.

### 2.3 Inclusion criteria and exclusion criteria

Consenting children and adults between the ages 4–88 years living within selected endemic communities, with hookworm monoinfection, and without any other chronic or allergic infections were recruited as cases while apparent healthy individuals without any parasitic infection were recruited as controls. However, children and adults who did not give informed consent, do not live in the selected communities or have any chronic infection or allergic infection were excluded.

### 2.4 Crude *N. americanus* egg antigen preparation

Eggs isolated from stools of *N. americanus* infected individuals were used for the crude antigen extraction. The eggs were suspended in 4°C 1X phosphate‐buffered saline (PBS) at a concentration of about 500 eggs/ml. A prechilled homogenizer was used to homogenize the eggs. The solution was boiled, frozen in liquid nitrogen, and thawed and homogenized three times. When approximately about 95% (or more) of the eggs were shredded/disrupted, the crude mixture was centrifuged at 4°C at 15,000 rpm for 60 min. The supernatant was collected and sterilized by passing it through a 0.2 µm filter. They were then aliquoted into 2 ml cryovial tubes and stored at −80°C.

### 2.5 PCR identification of hookworm species

Hookworm species identification was determined using genomic DNA (gDNA) extracted from purified hookworm egg samples of infected individuals using QIAamp DNA Stool Kit (QIAGEN).^21^, ^22^ Purified gDNA (20–40 ng) was used in PCR for the amplification of the internal transcribed region of ribosomal DNA.^23^ The PCR reaction contained the forward primer (NC2; 5′‐TTA GTT TCT TTT CCT CCG CT‐3′), with species‐specific reverse primers for *A. duodenale* (jmAD; 5′‐TGCGAA GTT CGC GTT CGC TGA GC‐3′) or *N. americanus* (jmNA; 5′‐CGTTAA CAT TGT ATA CCT GTA CAT AC‐3′) in separate reactions.^23^ The reaction mixtures also contained 1.25 mM each of deoxynucleotide triphosphate, 1 µ/L of the Taq DNA polymerase enzyme (Sigma‐Aldrich; Cat. # D1806‐250UN), in the reaction buffer. Negative (no template, nuclease‐free water) controls were included in all experiments. The PCR cycling conditions were, initial heating at 94°C for 5 min, followed by 40 cycles of denaturation at 94°C for 1 min, annealing at 55°C for 1 min, and extension at 72°C for 1 min, with a final elongation step at 72°C for 5 min. The amplified products were visualized and the sizes were determined by UV visualization after electrophoresis in a 2% ethidium bromide stained‐agarose gel. Products of the appropriate size (690 bp for *A. duodenale* and 870 bp for *N. americanus*) were considered positive compared to standard controls.

### 2.6 Measurement of IgE antibody against crude *N. americanus* egg antigens

Plasma antibodies to *N. americanus* egg antigens were estimated by modified quantitative enzyme‐linked immunosorbent assay (ELISA).^24^, ^25^, ^26^ Concisely, ELISA plates (Nunc, Maxisorp; Thermo Fisher Scientific) were coated overnight at 4°C with 3.0 µg/ml (100 µl/ml) of *N. americanus* crude egg antigen diluted in PBS buffer (137 mM NaCl, 2.7 mM KCl, 8.1 mM Na_2_HPO_4_, KH_2_PO_4_, pH 7.2–7.4). The plates were washed four times with washing buffer (PBS/0.1% Tween‐20; pH 7.2–7.4) and were then blocked for 1 h at room temperature (RT) with 200 µl of blocking buffer (PBS/0.1% Tween‐20/5% bovine serum albumin). The plates were tapped on a pad and washed four times in washing buffer (for each washing step, the plates were filled with washing buffer for 1 min before they were emptied). Individual and positive control serum samples were diluted 1:50 in PBS/0.1% Tween‐20/2.5% bovine serum albumin buffer, and 100 µl were added in duplicate to the respective wells, and plates were incubated for 2 h at RT. A pool of hyperimmune serum samples was twofold titrated downward with a starting dilution of 1:50 and 100 µl were added to each plate as a standard and also PBS buffer blank (serum dilution buffer) was added in duplicate to the wells. The plates were washed (4x) and 100 µl per well of alkaline phosphatase‐conjugated detection antibody in PBS/0.1% Tween‐20/2.5% bovine serum albumin buffer was added at the following dilutions 1: 1000 (Life Technologies; Cat # H15707) and incubated for 1 h at RT. The plates were washed, and treated with 3,3′,5,5′‐tetramethylbenzidine substrate (Kem‐En‐Tec Diagnosis A/S) and incubated for 30 min at RT in the dark until the color reaction was stopped with 0.2 M sulfuric acid (H_2_SO_4_). Optical density (OD) values were read at 450 nm with a reference wavelength of 570 nm with an automated ELISA reader (BioTek 405). The OD values obtained were converted into arbitrary units (AUs) using the four‐parameter curve fitting software (ADAMSEL, version 1.1 build 40 © 2009 EJ Remarque). The two‐time point samples for each individual were tested on the same ELISA plate to avoid differences that may have been due to interplate variations.

### 2.7 Blood eosinophil determination

The blood eosinophil levels were determined using a hematology analyzer (ABX Pentra 60C+; HORIBA Medical) by following the manufacturer's instructions.

### 2.8 Eotaxin (CCL11) and ECP measurements

Plasma samples collected from the participants were analyzed for the levels of eotaxin (CCL11) by sandwich ELISA using the DuoSet ELISA kit (R&D Systems Inc.) according to the manufacturer's instructions. ECP levels were also measured by ELISA using the MESACUP ECP Test Kit (MBL Co., Ltd.) following the manufacturer's guidelines. Briefly, plasma samples were mixed with an assay diluent and transferred to a 96‐well microplate pre‐coated with an anti‐human ECP antibody. After incubation and washing, 100 μl of horseradish peroxidase‐conjugated anti‐human ECP polyclonal antibody was added and followed with the substrate reagent tetramethylbenzidine/H_2_O_2_. The absorbance of each well was read at 450 nm in an ELISA plate reader (SpectraMax 340 PC; Molecular Devices). The ADAMSEL program was used to convert the OD values from the ELISA into ECP concentrations.

### 2.9 Statistical analyses

Statistical analyses were performed on the R language for statistical computing.^27^ Age was presented as median with interquartile ranges for both groups whilst gender was presented as frequencies with percentages. Distribution and levels of eosinophil‐mediated immune response markers were present by kernel density plot and violin plot respectively and subsequent Mann–Whitney *U* test. A multiple linear regression model was used to assess the association between eosinophil‐mediated immune response markers and hookworm intensity. The receiver operator characteristics (ROC) analysis from the pROC package in R^28^ was used to determine the diagnostic accuracies of the markers. *p‐*values less than 0.05 were considered statistically significant.

## 3 RESULTS

A total of 76 participants consisting of 40 hookworm‐positive patients (cases) and 36 hookworm negative (controls) were recruited, and their samples analyzed. The median age of hookworm positive participants was similar to that of hookworm negative controls (37.0 [20.5–46.8] vs. 27.0 [19.0–41.0] years, *p* = 0.235). There was no statistical difference in male and female proportions between the two groups (*p* = 0.864). Table 1 displays the sociodemographic characteristics of the study participants.

**Table 1:** Sociodemographic characteristics of the study participants

	Hookworm infection status		
Variable	Negative (*n* = 36)	Positive (*n* = 40)	*p*
Age	37.0 (20.5–46.8)	27.0 (19.0–41.0)	0.235
Gender			
Male	16 (44.4)	17 (42.5)	0.864
Female	20 (55.6)	23 (57.5)	0.864

**Figure 1:**
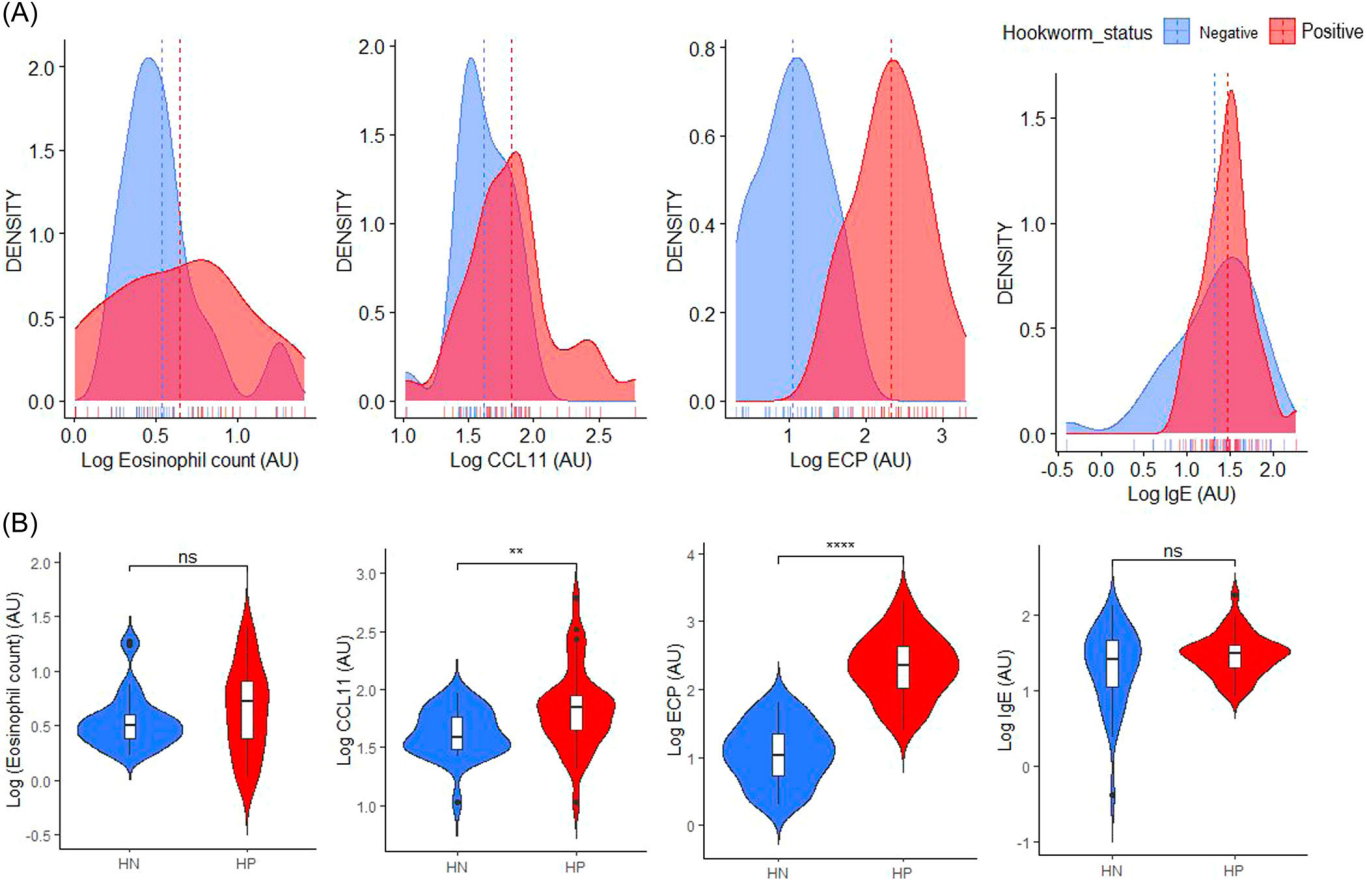
Levels of eosinophil‐mediated immune responses markers among study groups. Distribution (A) and violin plots (B) levels of eosinophil‐mediated immune responses markers among study groups. AU, arbitrary unit; CCL11, eotaxin; ECP, eosinophil cationic protein; HN, hookworm negative; HP, hookworm positive; IgE, immunoglobin E; ns, not significant.

The hookworm positive participants had significantly higher levels of ECP (2.34 [1.99–2.66] AU vs. 1.02 [0.71–1.37] AU, *p* < 0.0001) and CCL11 (1.85 [1.64–1.96], *p* < 0.001) compared to the hookworm negative controls. However, there was no significant difference in the eosinophil count and IgE between the two groups (*p* > 0.05) (Figure 1).

### 3.1 Association between eosinophil‐mediated immune responses markers and hookworm intensity

**Figure 2:**
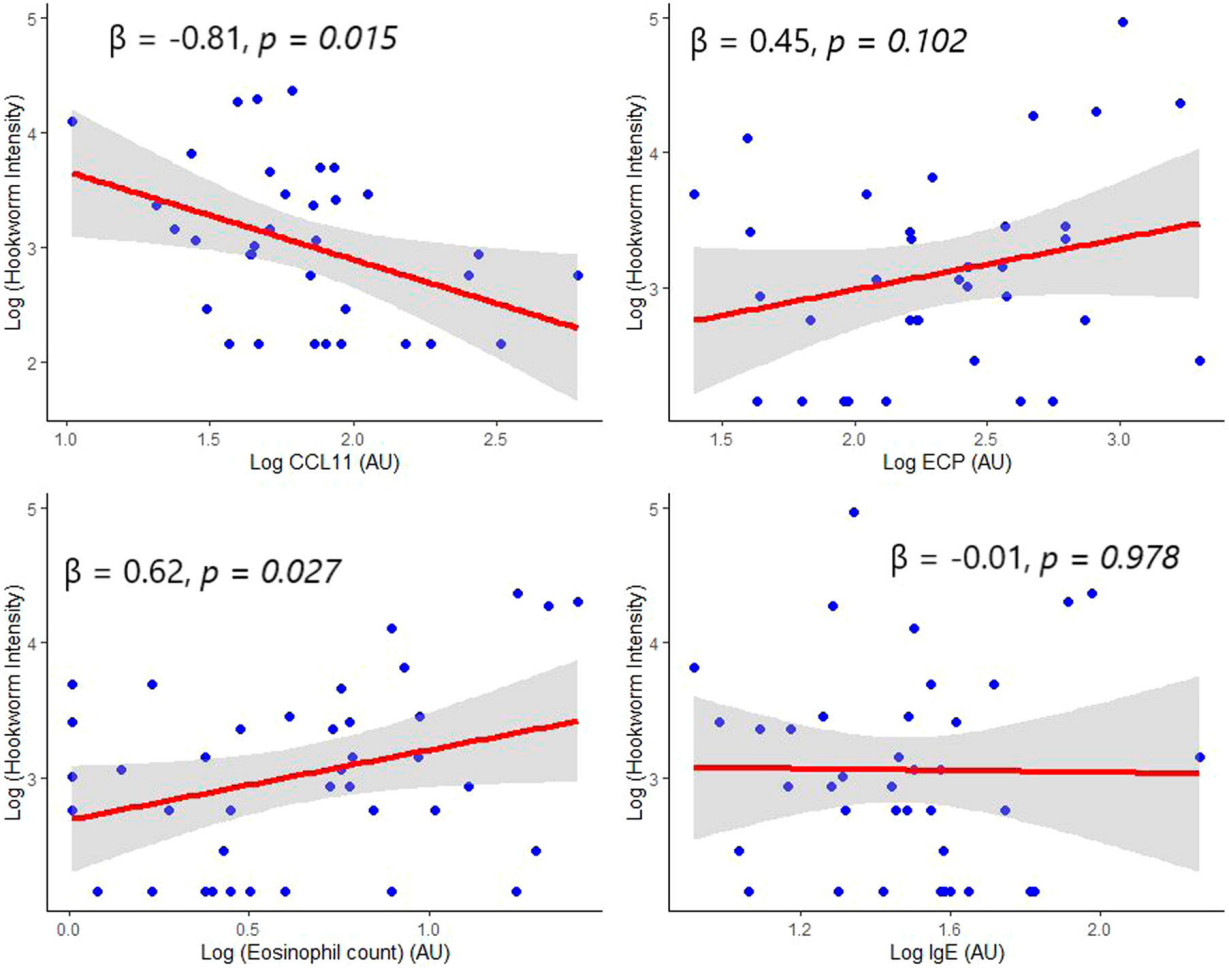
Association between eosinophil‐mediated immune responses markers and hookworm intensity. Model adjusted for age and sex. AU, arbitrary unit; CCL11, eotaxin; ECP, eosinophil cationic protein; IgE, immunoglobin E.

In a multivariate linear regression model, increasing CCL11 (*β* = −0.81, *p* = 0.015) was associated with a significant decrease in hookworm intensity. Again, increasing IgE (*β* = −0.01, *p* = 0.978) was associated with a slight decrease in hookworm intensity; however, the association was not statistically significant. In contrast, increasing eosinophil count (*β* = 0.62, *p* = 0.027) was associated with significant increase in hookworm intensity. Moreover, increasing ECP (*β* = 0.62, *p* = 0.027) was associated with an increase in hookworm intensity; however, this association was not statistically significant (Figure 2).

### 3.2 Diagnostic performance of eosinophil‐mediated immune responses markers in detecting hookworm infection

**Figure 3:**
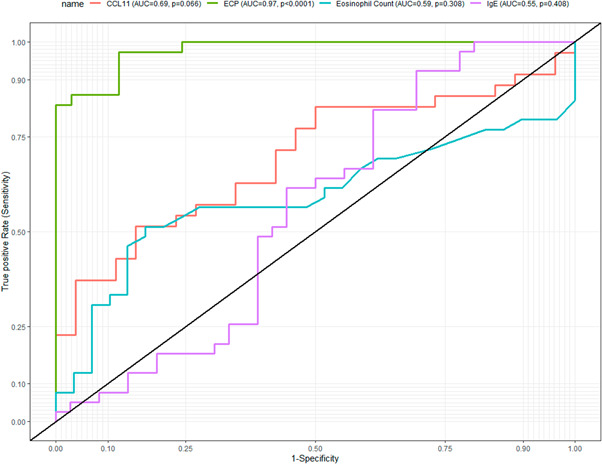
ROC curves of eosinophil‐mediated immune responses markers in detecting hookworm infection. AUC, area under the curve; CCL11, eotaxin; ECP, eosinophil cationic protein; IgE, immunoglobin E; ROC, receiver operator characteristics.

In a ROC analysis for detecting hookworm infection, ECP could significantly detect hookworm infection with a very high area under the curve (AUC = 0.97, *p* < 0.0001). However, eosinophil count (AUC = 0.59, *p* = 0.308), CCL11 (AUC = 0.69, *p* = 0.06), and IgE (AUC = 0.55, *p* = 0.408) count not significantly detect hookworm infection (Figure 3).

At a cutoff of 39.05, ECP was the best eosinophil‐mediated immune response marker for detecting hookworm infection with a sensitivity of 97.2%, specificity of 87.8%, a positive predictive value of 89.7%, and a negative predictive value of 96.6%. At a cutoff of 5.30, the eosinophil count was specific (82.7%) but less sensitive (51.2%). At a cutoff of 70.54, CCL11 was specific (84.6%) but less sensitive (51.4%). Moreover, at a cutoff of 11.52, IgE was sensitive (92.3) but less specific (30.5%). Table 2 displays the diagnostic performance of the eosinophil‐mediated immune response marker for detecting hookworm infection.

**Table 2:** Diagnostic performance of eosinophil‐mediated immune response marker for detecting hookworm infection

Marker	Cutoff	Sensitivity (95% CI)	Specificity (95% CI)	PPV	NPV	LR+	LR−
Eosinophil count	5.30	51.2 (36.2–66.1)	82.7 (64.8–92.7)	80.0.0	55.8	2.97	0.58
CCL11	70.54	51.4 (35.5–66.9)	84.6 (65.6–94.3)	81.8	56.4	3.34	0.57
ECP	**39.05**	**97.2 (84.3–100)**	**87.8 (71.9–95.6)**	**89.7**	**96.6**	**8.02**	**0.03**
IgE	11.52	92.3 (78.7–97.9)	30.5 (17.9‐47.0)	59	78.5	1.32	0.25

*Note*: At a cut‐off of 39.05, ECP was the best eosinophil‐mediated immune response marker for detecting hookworm infection with a sensitivity of 97.2%, specificity of 87.8%, a positive predictive value (PPV) of 89.7%, and a negative predictive value of 96.6%.

Abbreviations: CCL11, eotaxin; CI, confidence interval; ECP, eosinophil cationic protein; IgE, immunoglobin E; LR−, negative likelihood ratio; LR+, positive likelihood ratio; NPV, negative predictive value; PPV, positive predictive value.

## 4 DISCUSSION

The role of eosinophils and parasite‐killing antibody isotype IgE has been described in the control of hookworm infections.^29^ Here, we identified marked differences in eosinophil‐mediated immune responses between hookworm infected individuals and negative endemic controls. Specifically, we utilized a combination of plasma ECP, circulating eosinophil, CCL11, and IgE to identify and characterize immune responses in hookworm infection. We observed higher levels of ECP in hookworm‐infected individuals which confirms our previous finding.^20^ Hence, we report the diagnostic potential of plasma ECP in hookworm infection.

Several studies into the immunopathology of hookworm infection have previously reported higher ECP levels.^18^, ^29^, ^30^ ECP, as a cationic protein, is recruited to facilitate the disintegration of parasitic helminths. This may account for the extremely high plasma levels of ECP in hookworm patients. In concordance with our finding, earlier reports showed higher ECP levels in hookworm and other parasitic helminth infections when compared to both endemic and nonendemic controls.^31^ Additional findings showed elevated CCL11 in infected individuals, which had not been previously reported. CCL11, also known as eotaxin‐1, is a potent eosinophil chemoattractant that stimulates the release of eosinophils. The activation of eosinophils in response to hookworm infection leads to the killing of parasitic helminth by the release of its toxic granular constituents.^32^ This activity of host immune response may account for the high levels of CCL11 in individuals with hookworm infection observed in this study.

This study discovered that a rise in intensity of hookworm infection (EPG) causes a corresponding increase in eosinophil count whereas the CCL11 level decreases. This suggests the potential of either marker being used as treatment response biomarkers in the monitoring of hookworm infection intensity during therapy. Other studies have also found elevated eosinophil count in hookworm patients as well as other parasitic helminth infections.^6^, ^18^, ^31^, ^33^ It is possible that due to ongoing immune activation and release of eosinophil due to hookworm infection, individuals presenting with increased parasite load also undergo increased immune activity. CCL11, on the other hand, is known to activate eosinophils via a CCR3 pathway. Other studies have shown alternate recruitment mediators outside of CCL11 that induce eosinophil recruitment as well as the role of enzymatic inhibition on these chemoattractant mediators.^34^ It may be that with increased hookworm intensity, increased eosinophil levels were not via a CCL11‐induced pathway. The interplay of various eotaxins in the advent of eosinophilia in parasitic helminth infections needs to be clarified.

In this study, the use of hookworm crude antigens reduces the possible interference of other helminths in the observed patterns of eosinophil count and CCL11 with increasing hookworm intensity. However, the possible influence of secondary helminth infections on the levels of eosinophil‐mediated immune responses cannot be completely ruled out. It would be critical for further investigations using multiple helminth‐derived antigens to ascertain hookworm‐specific immune responses in infected individuals. Previous studies, including ours, found that increasing plasma ECP correlates with increasing hookworm infection intensity.^18^, ^20^, ^31^ Although the current study reported the same, this correlation did not reach the level of statistical significance.

Furthermore, we investigated if eosinophil‐mediated immune markers could discriminate hookworm infected individuals from negative endemic controls. Indeed, ROC analyses showed a strong capacity of ECP to distinguish infected persons from the control group. This finding confirmed our previous study which also showed the potential of ECP as a hookworm diagnostic biomarker.^20^ Although egg counts are the most standard method of detecting helminth infections, they have a number of flaws that make them less than ideal. They cannot detect infections that are immature since they are not laying eggs yet, and using a single stool sample can sometimes miss infections due to egg output inconsistency. As a result, biomarkers may be a stronger indicator than egg numbers. From the current study, infected persons with plasma ECP levels above 39.05 ng/ml would likely produce a positive microscopy result 96.6% of the time. On the other hand, the same threshold is likely to return a microscopy negative test as negative 89.7% of the time. Nevertheless, any effort to use ECP as a diagnostic marker for hookworm infection must take into account the impact of concurrent helminth infections on plasma ECP expression. As indicated earlier, the use of crude antigens confers some level of hookworm specificity in the measurement of ECP levels. However, the use of crude antigen is not exempt from cross‐reactions. One limitation of the current study is its relatively small sample size; however, it was good enough to make the immunological inference. Effective control and monitoring programs are crucial to achieving the World Health Organization 2030 global targets for soil‐transmitted helminthiases. Findings from this study serve as a platform for the development of accurate and efficient diagnostic biomarkers for hookworm infection and disease monitoring.

## 5 CONCLUSION

ECP is the best predictive eosinophil‐mediated immune response marker for hookworm infection, while CCL11 and eosinophil count better predict hookworm intensity. Serum ECP level may be a good biomarker of hookworm infection and intensity. Further multicentre investigations are needed to help improve eosinophil‐mediated immune response as markers predictive markers for hookworm diagnosis.

## AUTHOR CONTRIBUTIONS

**Samuel A. Sakyi**: Conceptualization; data curation; investigation; methodology; project administration; resources; supervision; validation; writing–original draft; and writing–review and editing. **Benjamin Amoani**: Conceptualization; data curation; formal analysis; resources; software; validation; writing–original draft; and writing–review and editing. **Stephen Opoku**: Data curation; formal analysis; investigation; methodology; software; validation; visualization; writing–original draft; and writing–review and editing. **Lawrence Dzata**: Conceptualization; data curation; investigation; methodology; project administration; resources; visualization; writing–original draft; and writing–review and editing. **Wilfred Aniagyei**: Data curation; funding acquisition; investigation; methodology; validation; visualization; and writing–review and editing. **Ebenezer Senu**: Conceptualization; data curation; formal analysis; validation; writing–original draft; and writing–review and editing. **Kwabena Dankwa**: Conceptualization; project administration; resources; software; supervision; and writing–review and editing. **Michael D. Wilson**: Conceptualization; funding acquisition; investigation; methodology; project administration; resources; supervision; and writing–review and editing.

## CONFLICT OF INTEREST

The authors declare no conflict of interest.

## ETHICS STATEMENT

Ethical approval was sought from the Ethical Review Committee of the Noguchi Memorial Institute for Medical Research (No. FWA#: 00001824). A thorough explanation of the study protocol and assurance of anonymity was made to the subjects. Written informed consent was also sought from participants. For children below 18 years, parental consent was sought. All procedures were carried out in accordance with appropriate guidelines and procedures. This study was conducted according to the strengthening of the reporting of observational studies in the epidemiology statement for reporting observational studies.

## TRANSPARENCY STATEMENT

The lead author affirms that this manuscript is an honest, accurate, and transparent account of the study being reported; that no important aspects of the study have been omitted; and that any discrepancies from the study as planned (and, if relevant, registered) have been explained.

## Data Availability

All data generated or analyzed during this study are included in this article and its Supporting Information files data and can be requested from the corresponding author.
